# Enhancing Fall Prevention in Public Senior Housing Through Smart Home Safety Technology

**DOI:** 10.1093/geroni/igaf122.1884

**Published:** 2025-12-31

**Authors:** Yong Kyung Choi, Patricia Karg, Portia Singh, Haomin Hu, Julie Faieta, Meredith Hughes, Jon Pearlman, Steven Handler

**Affiliations:** University of Pittsburgh, Pittsburgh, Pennsylvania, United States; University of Pittsburgh, Pittsburgh, Pennsylvania, United States; University of Pittsburgh, Pittsburgh, Pennsylvania, United States; University of Pittsburgh, Pittsburgh, Pennsylvania, United States; University of Pittsburgh, Pittsburgh, Pennsylvania, United States; University of Pittsburgh, Pittsburgh, Pennsylvania, United States; University of Pittsburgh, Pittsburgh, Pennsylvania, United States; University of Pittsburgh, Pittsburgh, Pennsylvania, United States

## Abstract

Falls are the leading cause of injury among older adults, accounting for over 32,000 deaths and three million emergency department visits annually in the U.S. Older adults in public housing face increased fall risks due to environmental hazards, limited accessibility modifications, and restricted access to preventive interventions. Despite the potential of smart home safety technologies to mitigate these risks, their adoption in public housing remains limited due to usability concerns, cost barriers, and lack of implementation strategies. This study aims to identify and evaluate smart home safety technologies that can reduce fall risks and enhance safety for older adults in public senior housing. Using a community-based participatory research approach, we engage residents and housing authorities to assess fall-related hazards, technology usability, and implementation feasibility. The study consists of three phases: (1) a needs assessment to identify key fall risk factors, existing mitigation strategies, and policy barriers; (2) expert evaluation and usability testing of smart home safety technologies with older adults in a controlled simulation lab; and (3) pilot implementation of a tailored smart home intervention in public housing units. Technologies under evaluation include motion-activated lighting, environmental sensors, wearable fall detection devices, and smart medication management tools. At this conference, we will present preliminary findings, providing insights into older adults’ perceptions of smart home safety technologies and the specific technologies identified and evaluated. This research aligns with national aging-in-place initiatives and has significant implications for housing policy, health equity, and digital inclusion.

